# Quantitative Flow Ratio Based on Murray Fractal Law: Accuracy of Single Versus Two Angiographic Views

**DOI:** 10.1016/j.jscai.2022.100399

**Published:** 2022-08-26

**Authors:** Daixin Ding, Shengxian Tu, Yunxiao Chang, Chunming Li, Bo Xu, William Wijns

**Affiliations:** a The Lambe Institute for Translational Medicine, Smart Sensors Laboratory and Curam, National University of Ireland Galway, Ireland; b Biomedical Instrument Institute, School of Biomedical Engineering, Shanghai Jiao Tong University, Shanghai, China; c Shanghai Pulse Medical Technology Inc., Shanghai, China; d Catheterization Laboratories, Fuwai Hospital, National Center for Cardiovascular Diseases, Chinese Academy of Medical Sciences and Peking Union Medical College, Beijing, China

**Keywords:** coronary angiography, coronary physiology, fractional flow reserve, quantitative flow ratio

## Abstract

**Background:**

Murray bifurcation fractal law–based quantitative flow ratio (QFR), namely, μQFR, is a novel method for the fast computation of fractional flow reserve (FFR) from a single angiographic view. We aimed to compare the diagnostic accuracy of computational QFR based on single vs 2 angiographic views in patients with intermediate coronary stenosis.

**Methods:**

The algorithm of μQFR was extended to develop a Murray law–based 3-dimensional (3D) μQFR from 2 angiographic projections. Patients with both angiographic views acquired according to the protocol-specified recommended views in the FAVOR (Functional Diagnostic Accuracy of Quantitative Flow Ratio in Online Assessment of Coronary Stenosis) II China study were included. μQFR was computed separately from the first (μQFR1) and second (μQFR2) angiographic projections, whereas the 3D-μQFR was computed based on both projections, all blinded to FFR data. Hemodynamically significant coronary stenosis was defined by wire-based FFR of ≤0.80.

**Results:**

Altogether, 280 vessels from 262 patients had 2 protocol-specified recommended angiographic views; μQFR1, μQFR2, and 3D-μQFR were successfully computed in all these vessels. The mean FFR was 0.82 ± 0.12. The vessel-level diagnostic accuracy for μQFR1, μQFR2, and 3D-μQFR to identify hemodynamically significant stenosis was 92.1% (95% CI, 89.0%-95.3%), 92.5% (95% CI, 89.4%-95.6%), and 93.2% (95% CI, 90.3%-96.2%), respectively, with similar areas under the receiver operating characteristic curve for μQFR1 (0.96, *P* < .001), μQFR2 (0.95, *P* < .001), and 3D-μQFR (0.95, *P* < .001). μQFR1 and μQFR2 had excellent correlation (*r* = 0.95) and agreement (mean difference = 0.00 ± 0.03).

**Conclusions:**

Computation of μQFR from a single angiographic view had comparably good diagnostic performance as 2-view 3D-μQFR in identifying hemodynamically significant coronary stenosis.

## Highlights

• 1- and 2-view Murray law-based quantitative flow ratio have comparable accuracy.
• Both recommended views are reliable for Murray law-based quantitative flow ratio.
• Murray law-based quantitative flow ratio can facilitate functional test in cath lab.

## Central Illustration

**Figure:**
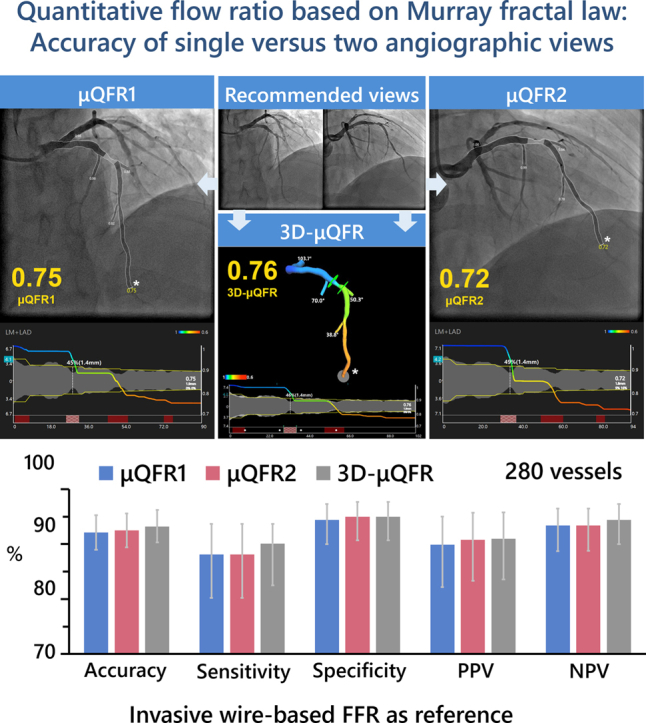
**Comparison of quantitative flow ratio based on Murray fractal law from single versus two angiographic views.** Upper: An example of μQFR1, μQFR2, and 3D-μQFR computation. Two protocol-specified recommended angiographic projections for an intermediate lesion in LAD were acquired. μQFR1 and μQFR2 were computed based on the first and second angiographic projections, separately, while 3D-μQFR was computed based on both projections. The lumen contours of LAD and its side branches were delineated. Computed pressure pullbacks were co-registered with lumen profile. The computed value was 0.75, 0.72, and 0.76 for μQFR1, μQFR2, and 3D-μQFR at the asterisk positions. Lower: Diagnostic performance of μQFR1, μQFR2, and 3D-μQFR in a total of 280 vessels with two protocol-specified angiographic views. μQFR1, μQFR2, and 3D-μQFR have comparably good diagnostic performance in predicting physiologically significant stenosis using wire-based FFR as reference. FFR, fractional flow reserve; LAD, left anterior descending artery; μQFR, Murray law-based quantitative flow ratio; NPV, negative predictive value; PPV, positive predictive value; 2D, 2-dimensional; 3D, 3-dimensional.

## Introduction

Quantitative flow ratio (QFR), a novel method for the fast computation of fractional flow reserve (FFR), has been extensively validated with clinically relevant diagnostic performance^1^, ^2^, ^3^, ^4^, ^5^, ^6^ and prognostic value.^7^ The recent FAVOR (Comparison of Quantitative Flow Ratio Guided and Angiography Guided Percutaneous Intervention in Patients with Coronary Artery Disease) III China trial also demonstrated that a QFR-guided strategy of vessel selection improved the 1-year clinical outcomes in patients undergoing percutaneous coronary intervention (PCI) compared with those undergoing standard angiography guidance.^8^

The computation of QFR hitherto required 3-dimensional (3D) vessel reconstruction from 2 angiographic projections with minimal overlap and foreshortening of the interrogated vessels in both projections, which decreases the feasibility of QFR computation, especially in retrospective studies without dedicated angiographic acquisition protocols.^5^ Furthermore, the first QFR solution assumes a linear tapering of the reference vessel diameter because the side branches were not included in the analysis. As such, the reconstruction of reference diameter function might be less accurate in the evaluation of bifurcation lesions, impairing the diagnostic accuracy of QFR in this stenosis subset.^9^

Recently, we validated a new method for computing QFR from a single angiographic view with step-down reference diameter across bifurcations.^10^ This Murray law–based QFR (μQFR) showed high diagnostic concordance with FFR in patients with intermediate coronary stenosis.^10^ It remains to be elucidated how to select the optimal angiographic view for the computation of single-view μQFR.

In this study, we extended the μQFR algorithm and developed a Murray law–based 3D-μQFR solution based on 2 angiographic projections. We aimed to compare the diagnostic accuracy of single-view μQFR and 2-view 3D-μQFR based on protocol-specified angiographic views.

## Methods

### Study population

This was a post hoc analysis based on the FAVOR (Functional Diagnostic Accuracy of Quantitative Flow Ratio in Online Assessment of Coronary Stenosis) II China study population (NCT03191708). The FAVOR II China study is a prospective, multicenter trial designed to evaluate the diagnostic accuracy of QFR in identifying hemodynamically significant coronary artery disease using wire-based FFR as the reference standard.^2^ A total of 332 interrogated vessels from 308 patients with suspected or known coronary artery disease who had at least 1 stenosis with percent diameter stenosis (DS%) between 30% and 90% in a vessel of ≥2 mm by visual assessment were enrolled at 5 hospitals in 3 major cities in China. The detailed study design, end points, inclusion and exclusion criteria have been published in the main study.^2^ The study complied with the Declaration of Helsinki and Good Clinical Practice guidelines of the China Food and Drug Administration. The study protocol was approved by the institutional review board, and all patients provided written informed consent.

The details of coronary angiography acquisition have been published in the main study^2^ and are specified in Supplemental Material. Of note, a table (Supplemental Table S1) with the recommended 2 optimal angiographic views specific for different types of interrogated vessel (left main and left main bifurcation, left anterior descending artery and diagonal artery, left circumflex and obtuse marginal, and right coronary artery) was provided to the operators before angiographic image acquisition.

All angiographic images were screened and analyzed at an academic core laboratory (CardHemo, Med-X Research Institute, Shanghai Jiao Tong University), without access to reference FFR values. Only vessels with both angiographic views acquired according to the protocol-specified recommended angiographic views (Supplemental Table S1) were included for analysis, whereas those with at least one of the 2 angiographic views not acquired according to the recommended projections were excluded. We assumed that an angiographic projection was acquired according to the recommended angiographic view if the actual acquisition angle was within 10° deviation from the recommended angiographic projection (Supplemental Figure S1).

### μQFR and 3D-μQFR analysis

The computations of μQFR and 3D-μQFR were performed using the AngioPlus Core software (version V2, Pulse Medical) by experienced analysts who had the official certification for μQFR and 3D-μQFR analysis.

The detailed methodology for single-view μQFR computation has been described elsewhere.^10^ In summary, the lumen contour of the interrogated epicardial coronary artery during contrast injection was delineated. Simultaneously, the contrast flow velocity was derived from the length of the vessel centerline divided by the contrast filling time, followed by conversion into hyperemic flow velocity.^11^ Subsequently, a frame with good contrast fill-in and full exposure of the lumen contour, especially at the stenotic segment, was selected as the analysis frame. The lumen boundaries of both the interrogated vessel and major side branches were delineated automatically. The reference vessel diameter was then reconstructed considering the step-down phenomenon across bifurcations based on the Murray bifurcation fractal law.^9^^,^^12^ Finally, the pressure drop was calculated based on the fluid dynamic equations with the above-mentioned hyperemic flow as the boundary condition,^10^ and μQFR was available for both the interrogated vessel and its side branches.

Afterwards, a second angiographic projection was introduced for the 3D reconstruction of the interrogated vessel. The lumen and reference vessel sizes were updated based on the 3D reconstruction data, and the 3D-μQFR was computed from the lumen and reference vessel sizes using the same fluid dynamic equations as in the 2-dimensional (2D) analysis. During the same calculation, the 2D and 3D quantitative coronary angiography (QCA) data were also available from the software.

After independent computation of the image-based indexes, the μQFR and 3D-μQFR values at the position that matched the location of the pressure sensor were used for comparison with the FFR value measured by the pressure wire.

For each interrogated vessel, the angiographic views acquired according to the first and second recommended angiographic views were termed the first angiographic projection and the second angiographic projection, respectively. Two analysts (X.L. and L.L.) performed the μQFR analysis separately using the first and second angiographic projection, with corresponding analysis results recorded as μQFR1 and μQFR2, respectively. A third analyst (B.W.) performed the 3D-μQFR analysis using both angiographic projections. Analyses of μQFR1, μQFR2, and 3D-μQFR were performed not only blinded to FFR values but also blinded to the other analysts’ results. To improve the comparability among μQFR1, μQFR2, and 3D-μQFR analyses, standardized analysis procedure was followed by all 3 analysts, including selection of the same start and end points for each interrogated vessel and use of the same key frame in angiographic projections for lumen delineation (Supplemental Material).

### FFR measurement

Details of FFR measurement have been published in the main study and are specified in Supplemental Material.

### Statistical analysis

Continuous variables were tested for normal distribution by the Kolmogorov-Smirnov test and are reported as mean ± SD if normally distributed or as median (quartiles) if non-normally distributed. Categorical variables are reported as counts (percentage). The Spearman correlation coefficient was used for correlation analysis. Bland-Altman analysis was used to test the agreement between different continuous variables. A comparison of the limits of agreement among μQFR1, μQFR2, and 3D-μQFR was performed using the F-test. A paired comparison of correlation coefficients was performed using the z test statistic. A comparison of continuous parameters derived from μQFR1 and μQFR2 analyses was performed using the Wilcoxon test or *t* test, as appropriate. The between-center heterogeneity for assessment of the mean agreement of μQFR1, μQFR2, and 3D-μQFR compared with FFR was tested by the *I*^*2*^ statistic. The area under the curve (AUC) by receiver operating characteristic (ROC) curve analysis by the Delong method was used to compare the accuracy of μQFR1, μQFR2, and 3D-μQFR in predicting FFR of ≤0.80. The Youden index was used as the criterion to determine the best cutoff values for μQFR1, μQFR2, 3D-μQFR, and corresponding QCA-derived DS%. All statistical analyses were performed using MedCalc version 14.12 (MedCalc Software) and Stata version 15.0 (StataCorp).

## Results

### Baseline clinical and lesion characteristics

**Figure. 1:**
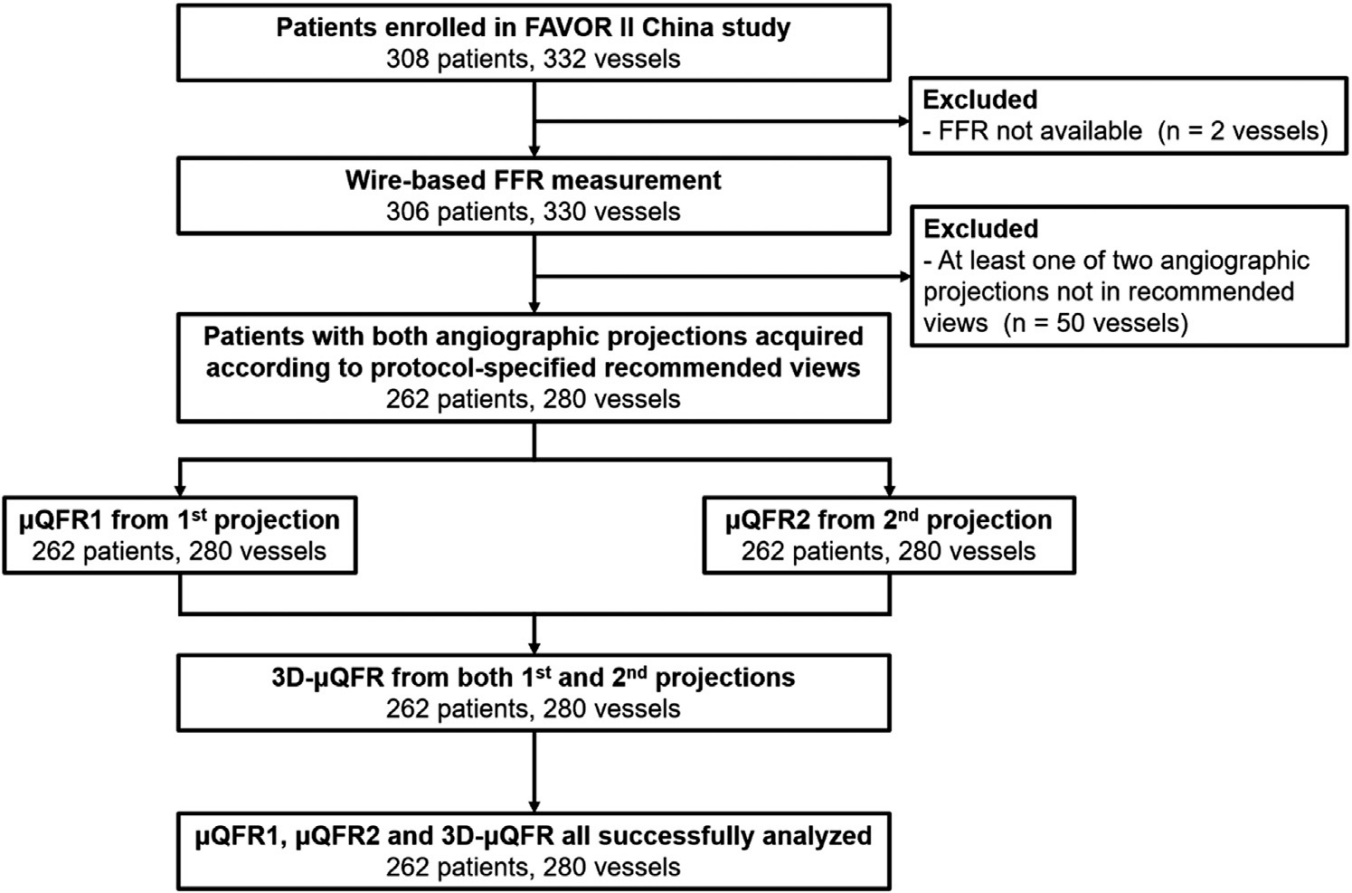
**Study flow chart.** A total of 330 vessels from 306 patients with both angiography and FFR from FAVOR II China study were screened. Fifty vessels were excluded because angiographic view(s) were not acquired according to the recommended projections. Subsequently, μQFR1 and μQFR2 were computed from the first and second projections, separately, while 3D-μQFR was computed based on both projections. Finally, μQFR1, μQFR2, and 3D-μQFR were successfully analyzed in 262 patients and 280 vessels. FFR, fractional flow reserve; μQFR, Murray law–based quantitative flow ratio; 3D, 3-dimensional.

The study flowchart is shown in Figure 1. A total of 330 vessels from 306 patients with both angiography and FFR data from the FAVOR II China study were screened in the core laboratory. Fifty vessels were excluded because at least one of the 2 angiographic views was not acquired according to the recommended angiographic projections. Eventually, 280 vessels from 262 patients with 2 protocol-specified recommended angiographic views constituted the population of this study. Specifically, the number of interrogated vessels from 5 participating centers was 35, 22, 126, 57, and 40, respectively.

The baseline clinical and lesion characteristics are listed in Tables 1 and 2. Angiographic projection angle distributions for all 280 interrogated vessels are shown in Supplemental Figure S1. A total of 159 (57%) interrogated vessels were left anterior descending arteries, and 75 (26.8%) vessels had bifurcation lesions.

**Table 1:** Baseline demographic characteristics.

Characteristics	N = 262
Age, y	61 ± 11
Female	71 (27%)
Body mass index, kg/m^2^	25 ± 3^a^
Diabetes mellitus	74 (28%)
Hypertension	156 (60%)
Hyperlipidemia	111 (42%)
Current smoker	80 (31%)
Family history of CAD	37 (14%)
Previous myocardial infarction	43 (16%)
Previous PCI	57 (22%)
Previous CABG	1 (0.4%)
Clinical presentation	
Silent ischemia	29 (11%)
Stable angina pectoris	67 (26%)
Unstable angina pectoris	154 (59%)
Acute myocardial infarction within 1 month	12 (5%)

Values are presented as mean ± SD or counts (percentage).

CABG, coronary artery bypass graft; CAD, coronary artery disease; PCI, percutaneous coronary intervention.

a n = 258.

**Table 2:** Baseline vessel characteristics.

Characteristics	280 Vessels
Interrogated vessel	
Left main coronary artery	1 (0.4%)
Left anterior descending artery	158 (56%)
Diagonal branch	1 (0.4%)
Left circumflex artery	48 (17%)
Obtuse marginal branch	4 (1%)
Ramus intermediate	1 (0.4%)
Right coronary artery	73 (26%)
Posterior descending artery	1 (0.4%)
Posterolateral branch	1 (0.4%)
FFR	
Mean ± SD	0.82 ± 0.12
Median (quartiles)	0.85 (0.77, 0.91)
μQFR1	
Mean ± SD	0.83 ± 0.12
Median (quartiles)	0.86 (0.76, 0.91)
μQFR2	
Mean ± SD	0.83 ± 0.12
Median (quartiles)	0.86 (0.76, 0.92)
3-D μQFR	
Mean ± SD	0.83 ± 0.12
Median (quartiles)	0.86 (0.77, 0.92)

Values are presented as mean ± SD, median (quartiles), or counts (percentage).

FFR, fractional flow reserve; SD, standard deviation; μQFR, Murray law–based quantitative flow ratio; 3D, 3 dimensional.

μQFR1 based on the first angiographic projection, μQFR2 based on the second angiographic projection, and 3D-μQFR based on 2 angiographic projections were successfully analyzed in all 280 vessels. FFR had a mean value of 0.82 ± 0.12 and a median value of 0.85 (0.77, 0.91). FFR of ≤0.80 was identified in 101 (36.1%) vessels (Supplemental Figure S2).

### Correlation and agreement with FFR

**Figure. 2:**
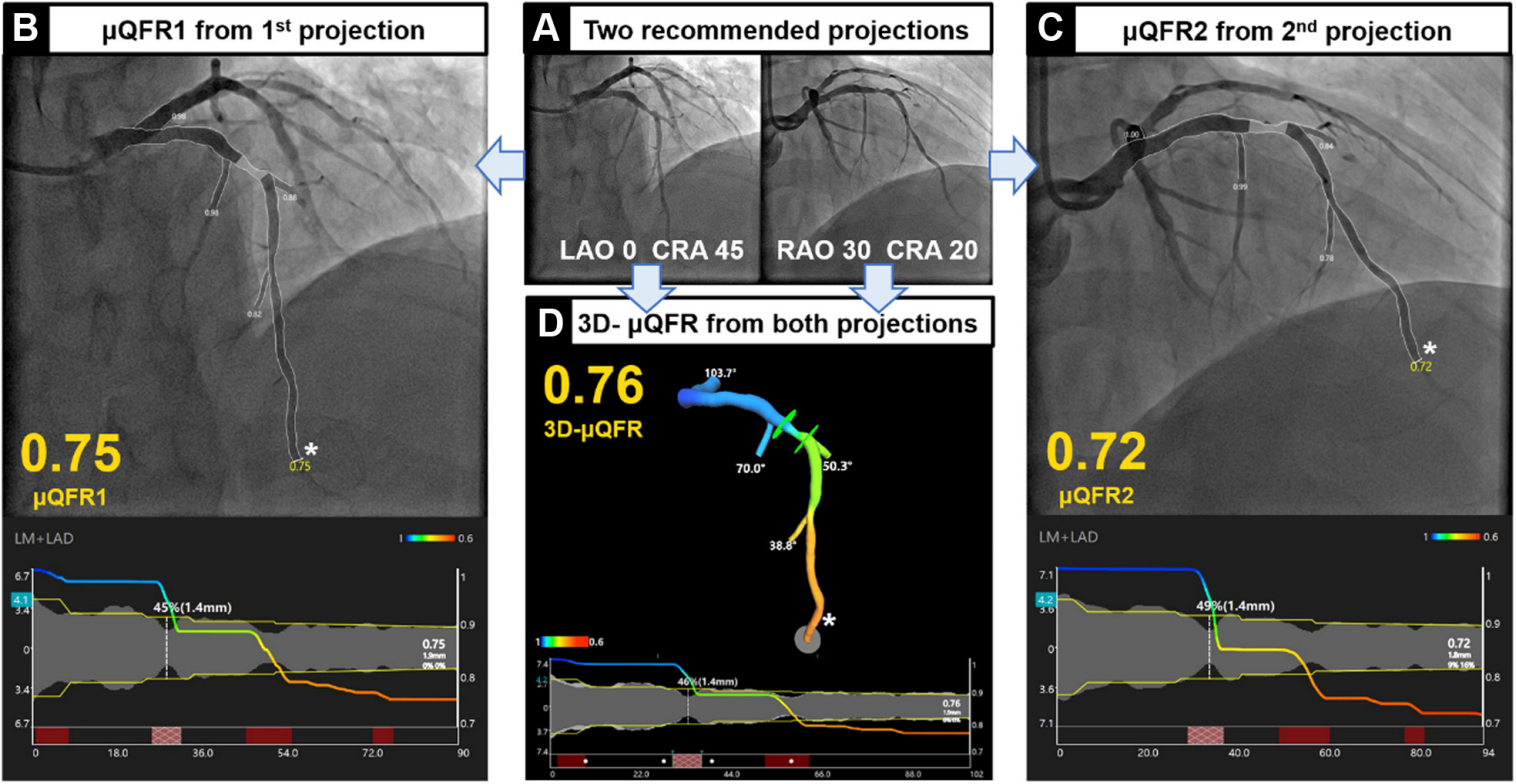
**Representative examples ofμQFR1, μQFR2, and 3D-μQFRcomputation.** (A) Two protocol-specified recommended angiographic projections for an intermediate lesion in the LAD. (B) μQFR1 computation based on the first angiographic projection. The lumen contours of the LAD and its side branches were automatically delineated and superimposed on the angiographic images. Lower panel shows the coregistration between lumen size and μQFR pullback at every position along the LAD. The computed μQFR1 at the asterisk position was 0.75. (C) μQFR2 computation based on the second angiographic projection. The computed μQFR2 at the asterisk position was 0.72. (D) 3D-μQFR computation based on both angiographic projections. The lumen contours of the LAD and its side branches were reconstructed in 3 dimensions. The computed 3D-μQFR at the asterisk position was 0.76. LAD, left anterior descending artery; LAO, left anterior oblique; μQFR, Murray law–based quantitative flow ratio; RAO, right anterior oblique; CRA, cranial; 3D, 3-dimensional.

Representative examples of μQFR1, μQFR2, and 3D-μQFR computation are shown in Figure 2. The median values for μQFR1, μQFR2, and 3D-μQFR were 0.86 (0.76, 0.91), 0.86 (0.76, 0.92), and 0.86 (0.77, 0.92), respectively. The mean values for μQFR1, μQFR2, and 3D-μQFR were all 0.83 ± 0.12. μQFR1 of ≤0.80, μQFR2 of ≤0.80, and 3D-μQFR of ≤0.80 were identified in 99 (35.4%), 98 (35.0%), and 100 (35.7%) vessels (Supplemental Figure S2).

**Figure. 3:**
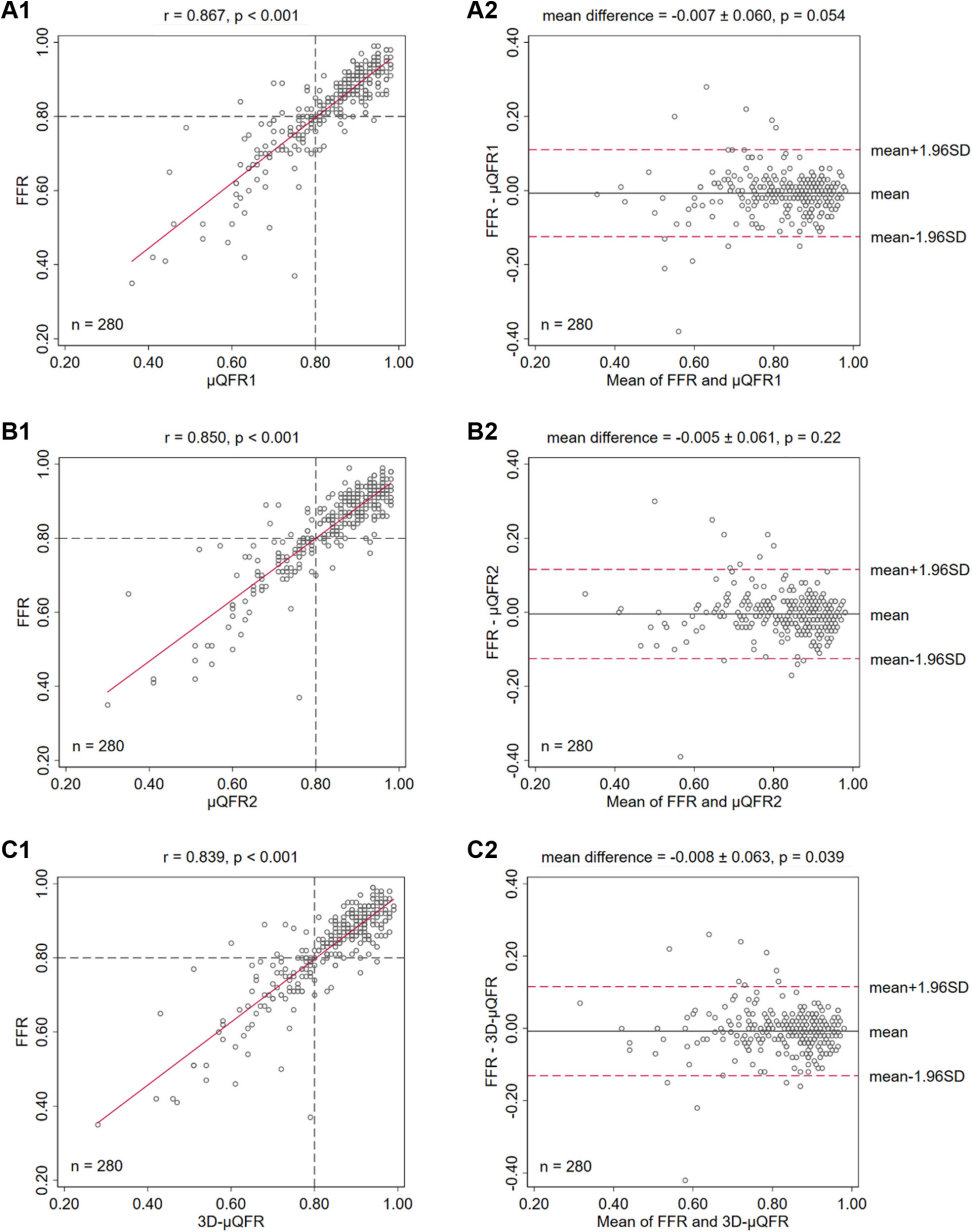
**Correlation and agreement of μQFR1, μQFR2, and 3D-μQFR related to wire-based FFR.** The correlation and agreement with FFR were comparable for μQFR1, μQFR2, and 3D-μQFR. FFR, fractional flow reserve; μQFR, Murray law–based quantitative flow ratio; 3D, 3-dimensional.

Figure 3 shows the correlation and agreement of μQFR1, μQFR2, and 3D-μQFR related to wire-based FFR. The correlation with FFR was comparable for μQFR1 (*r* = 0.87, *P* < .001), μQFR2 (*r* = 0.85, *P* < .001), and 3D-μQFR (*r* = 0.84, *P* < .001). The agreement with FFR was also comparable for μQFR1 (mean difference = −0.01 ± 0.06, *P* = .05), μQFR2 (mean difference = −0.00 ± 0.06, *P* = .22), and 3D-μQFR (mean difference = −0.01 ± 0.06, *P* = .04). μQFR1 and μQFR2 had excellent correlation (*r* = 0.95, *P* < .001) and agreement (mean difference = 0.00 ± 0.03, *P* = .23).

As to the between-center heterogeneity, the *I*^*2*^ statistic for assessment of the mean agreement with FFR for μQFR1, μQFR2, and 3D-μQFR were all 0.00% (*P* = .99), indicating that the between-center variance component was small enough to be ignored for paired comparison of mean difference.

### Diagnostic performance of μQFR1, μQFR2, and 3D-μQFR

Using a cutoff value of ≤0.80 for FFR to identify hemodynamically significant stenosis, the vessel-level diagnostic accuracy of μQFR1, μQFR2, and 3D-μQFR was comparable: 92.1% (95% CI, 89.0%-95.3%), 92.5% (95%CI, 89.4%-95.6%), and 93.2% (95% CI, 90.3%-96.2%), respectively (Table 3 and Supplemental Table S2). Most discordant cases with FFR were left anterior descending artery (LAD) lesions (Supplemental Tables S3-S5). The diagnostic accuracy was similar among different interrogated vessels for μQFR1, μQFR2, and 3D-μQFR (Supplemental Tables S6-S8). The diagnostic accuracy was similar between bifurcation and nonbifurcation lesions for μQFR1, μQFR2, and 3D-μQFR (Supplemental Table S9).

**Table 3:** Diagnostic performances of μQFR1, μQFR2, and 3D-μQFR of ≤0.80 in predicting FFR of ≤0.80.

	μQFR1 ≤ 0.80	μQFR2 ≤ 0.80	3D-μQFR ≤ 0.80
Accuracy, % (95% CI)	92.1 (89.0-95.3)	92.5 (89.4-95.6)	93.2 (90.3-96.2)
Sensitivity, % (95% CI)	88.1 (80.2-93.7)	88.1 (80.2-93.7)	90.1 (82.5-93.7)
Specificity, % (95% CI)	94.4 (90.0-97.3)	95.0 (90.7-97.7)	95.0 (90.7-97.7)
PPV, % (95% CI)	89.9 (82.2-95.0)	90.8 (83.3-95.7)	91.0 (83.6-95.8)
NPV, % (95% CI)	93.4 (88.7-96.5)	93.4 (88.8-96.5)	94.4 (90.0-97.3)
Positive LR (95% CI)	15.8 (8.6-28.9)	17.5 (9.2-33.3)	17.9 (9.4-34.0)
Negative LR (95% CI)	0.13 (0.07-0.2)	0.13 (0.07-0.2)	0.10 (0.06-0.2)
AUC (95% CI)	0.96 (0.93-0.98)	0.95 (0.92-0.98)	0.95 (0.92-0.97)
Optimal cutoff by Youden index	≤0.81	≤0.80	≤0.80

AUC, area under the ROC curve; FFR, fractional flow reserve; LR, likelihood ratio; NPV, negative predictive value; PPV, positive predictive value; ROC, receiver operating characteristic; μQFR, Murray law–based quantitative flow ratio; 3D, 3-dimensional.

**Figure. 4:**
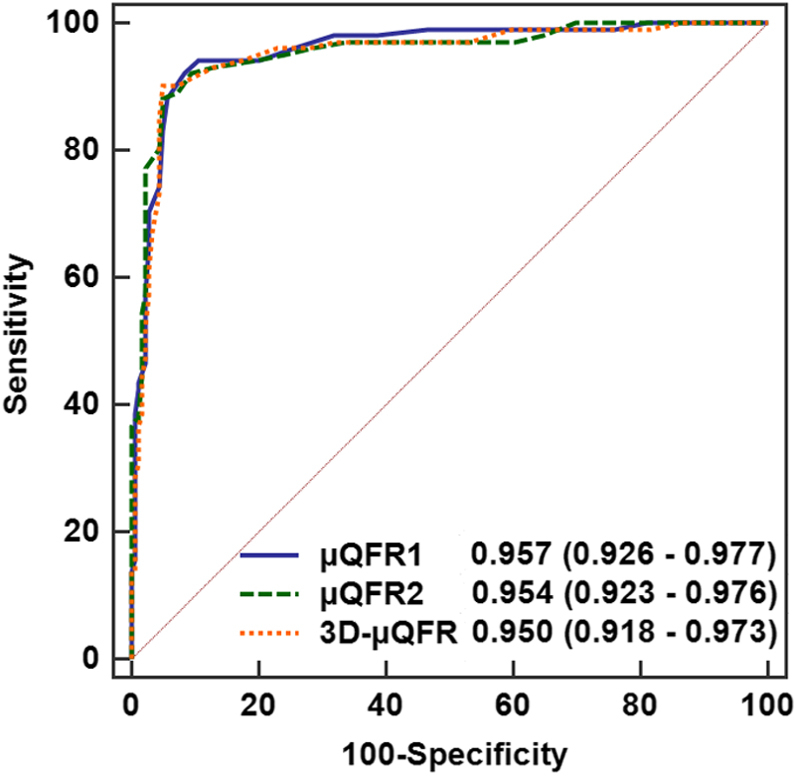
**Area under the receiver operating characteristic curve of μQFR1, μQFR2, and 3D-μQFR using FFR asthereference standard.** AUC was similar for μQFR1, μQFR2, and 3D-μQFR, using FFR as the reference standard, with no statistically significant difference for paired comparison. AUC, area under the receiver operating characteristic curve; FFR, fractional flow reserve; μQFR, Murray law–based quantitative flow ratio; 3D, 3-dimensional.

The AUC was similar for μQFR1 (0.96; 95% CI, 0.93-0.98; *P* < .001), μQFR2 (0.95; 95% CI, 0.92-0.98; *P* < .001), and 3D-μQFR (0.95; 95% CI, 0.92-0.97; *P* < .001), using FFR as the reference standard, with no statistically significant difference for paired comparison (Figure 4). Additional results of the diagnostic performance are listed in Table 3.

**Central Illustration:**
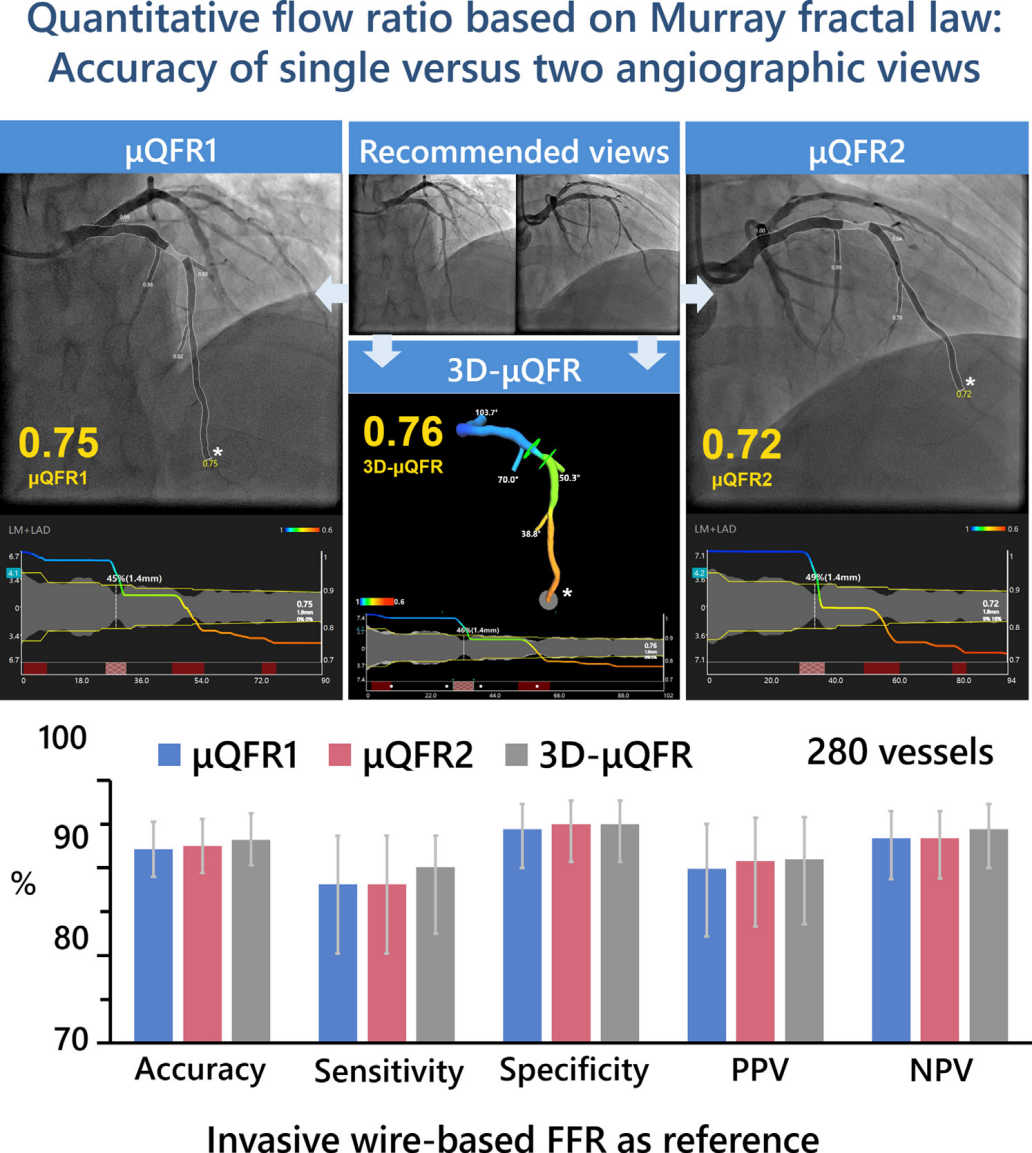
**Comparison of quantitative flow ratio based on Murray fractal law from single versus two angiographic views**. Upper: An example of μQFR1, μQFR2, and 3D-μQFR computation. Two protocol-specified recommended angiographic projections for an intermediate lesion in LAD were acquired. μQFR1 and μQFR2 were computed based on the first and second angiographic projections, separately, while 3D-μQFR was computed based on both projections. The lumen contours of LAD and its side branches were delineated. Computed pressure pullbacks were co-registered with lumen profile. The computed value was 0.75, 0.72, and 0.76 for μQFR1, μQFR2, and 3D-μQFR at the asterisk positions. Lower: Diagnostic performance of μQFR1, μQFR2, and 3D-μQFR in a total of 280 vessels with two protocol-specified angiographic views. μQFR1, μQFR2, and 3D-μQFR have comparably good diagnostic performance in predicting physiologically significant stenosis using wire-based FFR as reference. FFR, fractional flow reserve; LAD, left anterior descending artery; μQFR, Murray law-based quantitative flow ratio; NPV, negative predictive value; PPV, positive predictive value; 2D, 2-dimensional; 3D, 3-dimensional.

μQFR1, μQFR2, and 3D-μQFR values were concordant (all ≤0.80 or all >0.80) in 271 of 280 vessels. The 9 vessels with discordances among μQFR1, μQFR2, or 3D-μQFR values had a mean FFR of 0.80 ± 0.04 and median FFR of 0.80 (0.80, 0.82), with case-specific information shown in Supplemental Table S10.

The diagnostic performance of DS% of ≥50% derived from QCA in the 2 angiographic views and in 3D angiographic reconstruction was also comparable: 75% (95% CI, 70%-80%), 76% (95% CI, 71%-81%), and 74% (95% CI, 68%-79%), respectively (Supplemental Tables S2 and S11).

## Discussion

In the present study, we extended a single angiographic view–based μQFR to the development of 3D-μQFR based on 2 angiographic views (Central Illustration). In 280 vessels of 262 patients with 2 angiographic views acquired by protocol-specified recommended views in the FAVOR II China study, we found the following: (1) both single-view μQFR and 2-view 3D-μQFR had high feasibility when the 2 angiographic views were acquired according to the acquisition protocol; (2) both single-view μQFR and 2-view 3D-μQFR had good correlation and agreement with wire-based FFR; (3) single-view μQFR and 2-view 3D-μQFR had comparably good diagnostic performance in predicting hemodynamically significant stenosis defined by FFR of ≤0.80; and (4) either of the 2 protocol-specified acquisition views was reliable to compute μQFR.

The results of this study expanded on findings from the first validation study of μQFR,^10^ in which μQFR was successfully computed from a single angiographic view in all 330 vessels from 306 patients in the FAVOR II China study. For each interrogated vessel with 2 angiographic views that were initially selected for QFR analysis in the FAVOR II China study, the one with minimal overlap and foreshortening was designated as the “optimal angiographic view,” whereas the other was defined as the “suboptimal angiographic view.” It showed that μQFR computed from the optimal angiographic view had vessel-level diagnostic accuracy of 93.0% in predicting FFR of ≤0.80, whereas the use of suboptimal angiographic projection decreased the diagnostic accuracy of μQFR to 88.2% (AUC = 0.97 vs 0.92, difference = 0.05, *P* < .001).^10^ This emphasized the impact of angiographic projections on the precision of single-view μQFR. Nevertheless, the selection of the optimal angiographic view based on the amount of overlap and foreshortening was subjective. Whether it is reliable to use protocol-specified fixed angiographic views for μQFR computation is unknown. The present study was the first attempt to compare the diagnostic performance of single-view μQFR vs 2-view μQFR in a subgroup of the FAVOR II China population with 2 protocol-specified recommended angiographic views. We observed that single-view μQFR and 2-view 3D-μQFR had comparable diagnostic performance when protocol-specified recommended views were used. In theory, the 3D angiographic reconstruction based on 2 angiographic views can provide a more accurate geometrical model for the computation of FFR. However, this is valid only if both the angiographic views are optimal. In practice, it might be difficult to obtain a second optimal view in some patients, especially in vessels with complex anatomy. Therefore, the delineation of lumen contours in the second suboptimal angiographic view might be less accurate, resulting in suboptimal 3D reconstruction and μQFR computation. This was further confirmed by the comparable diagnostic accuracy of DS% derived from 2D-QCA (75%) and 3D-QCA (74%) in this study.

One vital concern is the possible discordance between single-view and 2-view–based QFR evaluations. In the current study, 9 of 280 vessels had discordant μQFR1, μQFR2, and 3D-μQFR results using the binary cutoff of 0.80. Of note, most of the binary mismatches (treat or defer) among μQFR1, μQFR2, and 3D-μQFR were cases close to the binary diagnostic cutoff point of 0.80, in which the benefit from revascularization was not demonstrated compared with deferred patients.^13^ In the remaining cases of the current population, excellent concordance was seen between μQFR and 3D-μQFR.

How to acquire the optimal angiographic projection for a target vessel or lesion is a critical issue.^14^ The ideal solution is to tailor the angiographic acquisition to individual vessel anatomy in the planning stage. Multiple attempts have been made to explore solutions for this purpose.^15^, ^16^, ^17^ Kočka et al^17^ defined the optimal fluoroscopic viewing angles for ostial coronary arteries and bifurcations using 3D coronary computed tomography angiography data. However, optimal viewing angles with minimal overlap and foreshortening of the target lesion(s) are highly variable among patients, and not all solutions are practical or achievable because of the physical limitation of the existing C-arm.^17^ In the current study, μQFR assessment based on either of the 2 protocol-specified recommended angiographic views had good diagnostic accuracy. This indicates the possibility of using these recommended views as the default views for μQFR assessment. It has several advantages: (1) to standardize and simplify clinical flow; and (2) to reduce the need for additional angiographic projection acquisitions and associated contrast dose, radiation exposure, and procedural time. Inevitably, because of the variability of individual coronary anatomy, the fixed views, derived from population-averaged practice, are not ideal for all individuals. If the 2 recommended projections are both suboptimal with no acceptable exposure of target lesions, additional angiographic projection(s) might be needed at the discretion of the operator.

Of note, we would like to emphasize that the excellent diagnostic accuracy of both single-view μQFR (92.1% and 92.5% for either angiographic view) and 3D-μQFR (93.2%) in this study was achieved based on protocol-specified recommended angiographic views that were prospectively acquired and with dedicated core laboratory analysis procedure. In clinical routine, less satisfactory results (Supplemental Table S12) are possible because of any or a combination of the following caveats, including unstandardized acquisition protocols, suboptimal angiographic projections or image quality, and failure to follow the standard operating procedure or to pinpoint the precise location at the interrogated vessel for comparison with the reference standard.

### Clinical perspectives

The overall feasibility and diagnostic accuracy of QFR computation depends on the image quality of the acquired angiographic projections (Supplemental Table S12). Decreased feasibility has frequently been reported in retrospective studies, mainly because of the lack of 2 adequate angiographic views.^5^ Additionally, the conventional QFR solution does not take side branches into consideration and might reduce the accuracy in the evaluation of bifurcation lesions.^9^

The results of this study indicate that μQFR based on a single angiographic projection is further anticipated to overcome the limitations of 2 views, with improved feasibility and maintained diagnostic accuracy. Particularly, in patients with tortuous, sequential, or diffuse lesions where it is not always possible to obtain 2 angiographic views, both with good exposure of all interrogated lesions, single-view μQFR will have better analyzability. It is possible to use the protocol-specified recommended angiographic views as the default views for μQFR assessment for further simplification of clinical flow. The use of artificial intelligence-facilitated automatic lumen contouring and frame counting greatly reduced the analysis time (average analysis time was 67 ± 22 seconds)^10^ for μQFR, with an anticipated shortened training cycle and improved reproducibility. Furthermore, because the computational QFR values for both the major epicardial coronary arteries and the side branches can be simultaneously obtained, μQFR is promising in the evaluation of bifurcation lesions. Meanwhile, 3D vessel reconstruction by 3D-μQFR could be of interest to quantify highly eccentric lesions, to provide the best viewing angles, and for accurate stent sizing.

The simplicity and straightforward procedure make it promising for the 2 technologies to be incorporated into clinical routine and improve the adoption of physiological assessment in the catheterization laboratory. Future validation of the feasibility and diagnostic performance of online μQFR and 3D-μQFR is warranted. Future studies investigating the usefulness of μQFR and 3D-μQFR in bifurcation lesions are welcome.

### Limitations

Our study is based on a population with chronic coronary syndromes or unstable angina. Therefore, the application of study results to patients with acute myocardial infarction needs further investigation. In the FAVOR II China study, bifurcation lesions with Medina 1,1,1 and 1,0,1 were excluded. Therefore, the usefulness of μQFR in these subsets of bifurcation lesions warrants further investigation. In addition, although μQFR can be used to assess the physiological significance of all side branches, wire-based FFR was measured predominantly in the main vessels in the FAVOR II China study. Therefore, further studies to validate the diagnostic accuracy of μQFR in side branches are warranted.

Only 1 left main bifurcation lesion acquired using protocol-specified angiographic views was included in the present study; therefore, the comparison between μQFR and 3D-μQFR in left main bifurcation lesions needs further investigation.

The FAVOR II China study did not enroll patients with coronary artery anomalies. Therefore, the generalization of the current findings to patients with coronary artery anomalies should be cautious. The use of coronary computed tomography angiography in identifying the optimal viewing angles for these patients could be useful.^17^

## Conclusions

The computation of μQFR from a single angiographic view and 3D-μQFR from 2 angiographic views had high feasibility when the angiographic views were acquired according to the recommended acquisition protocol. Single-view μQFR and 2-view 3D-μQFR had comparably good diagnostic performance in identifying hemodynamically significant coronary stenosis defined by FFR of ≤0.80. Either of the 2 protocol-specified acquisition views was reliable to compute μQFR.
