# Supplementary material for: Quantitative Flow Ratio Based on Murray Fractal Law: Accuracy of Single Versus Two Angiographic Views
Source: J Soc Cardiovasc Angiogr Interv. 2022 Aug 26;1(5):100399. doi: 10.1016/j.jscai.2022.100399 (PMC11307523; doi:10.1016/j.jscai.2022.100399)
Supplement: Supplementary Material [file mmc1.docx]

SUPPLEMENTAL MATERIAL

Quantitative flow ratio based on Murray fractal law: Accuracy of single versus two angiographic views

**CONTENTS**

[SUPPLEMENTAL METHODOLOGY 2](#_Toc108893248)

[Coronary angiography acquisition ^[1]^ 2](#_Toc108893249)

[FFR measurement ^[1]^ 2](#_Toc108893250)

[Improve the comparability among μQFR1, μQFR2, and 3D-μQFR analyses 3](#_Toc108893251)

[SUPPLEMENTAL FIGURES 4](#_Toc108893252)

[Supplemental Figure S1. Angiographic projection angle distribution. 4](#_Toc108893253)

[Supplemental Figure S2. Distribution histograms of FFR, μQFR1, μQFR2, and 3D-μQFR. 5](#_Toc108893254)

[SUPPLEMENTAL TABLES 6](#_Toc108893255)

[Supplemental Table S1. Recommended optimal angiographic projections in FAVOR II China 6](#_Toc108893256)

[Supplemental Table S2. Diagnostic concordance for predicting FFR ≤0.80 7](#_Toc108893257)

[Supplemental Table S3. Discordances between μQFR1 and FFR using 0.80 as the cutoff value 8](#_Toc108893258)

[Supplemental Table S4. Discordances between μQFR2 and FFR using 0.80 as the cutoff value 9](#_Toc108893259)

[Supplemental Table S5. Discordances between 3D-μQFR and FFR using 0.80 as the cutoff value 10](#_Toc108893260)

[Supplemental Table S6. Diagnostic accuracy of μQFR1 for predicting FFR ≤0.80 11](#_Toc108893261)

[Supplemental Table S7. Diagnostic accuracy of μQFR2 for predicting FFR ≤0.80 12](#_Toc108893262)

[Supplemental Table S8. Diagnostic accuracy of 3D-μQFR for predicting FFR ≤0.80 13](#_Toc108893263)

[Supplemental Table S9. Diagnostic accuracy for predicting FFR ≤0.80 in bifurcation lesions 14](#_Toc108893264)

[Supplemental Table S10. Discordances among μQFR1, μQFR2, and 3D-μQFR 15](#_Toc108893265)

[Supplemental Table S11. Diagnostic performance of DS% in predicting FFR ≤0.80 16](#_Toc108893266)

[Supplemental Table S12. Summary of key QFR validation studies 17](#_Toc108893267)

[REFERENCES 22](#_Toc108893268)

# SUPPLEMENTAL METHODOLOGY

## Coronary angiography acquisition ^[1]^

Angiographic images were recorded at 15 frames/s by flat-panel angiographic systems (AXIOM Artis, Siemens Healthcare, Erlangen, Germany; Innova, GE, Wauwatosa, Wisconsin; AlluraXper, Philips, Amsterdam, the Netherlands; INTEGRIS Allura, Philips). A table (Table S1) with recommended optimal angiographic views for different types of interrogated vessel (1. Left main and left main bifurcation, 2. left anterior descending artery and diagonal artery, 3. left circumflex and obtuse marginal, and 4. right coronary artery) was provided to the operators prior to angiographic image acquisition. Injection of the contrast medium was performed manually with a forceful and stable injection or by the pump at a rate of approximately 4 ml/s.

## FFR measurement ^[1]^

All FFR measurements were performed by using RadiAnalyzer Xpress instrument and Certus pressure wire (St. Jude Medical, St. Paul, Minnesota). Hyperemia was induced by intravenous administration of adenosine-50-triphosphate (ATP) at ≥160 μg/l/min. Pressure data were recorded for at least 3 s of stable value before ATP administration and for at least 10 s of stable value during hyperemia. The pressure sensor was returned to the guiding catheter tip to exclude pressure drift. A limited pressure drift within 0.05 during hyperemia was accepted; otherwise, the procedure was repeated. Notably, for FFR values between 0.75 and 0.85, only a smaller drift of 0.02 was considered acceptable.

## Improve the comparability among μQFR1, μQFR2, and 3D-μQFR analyses

To improve the comparability among μQFR1, μQFR2, and 3D-μQFR analyses, for each interrogated vessel, the key angiographic frame selected, and the start and end points of the interrogated vessel identified for subsequent analysis, were kept the same.

Before μQFR1, μQFR2, and 3D-μQFR analyses commence, a “screener” (not any of the three analysts) with 3-year experience in angiographic image interpretation screened both angiographic projections of each interrogated vessel, identified and recorded the following information: 1) the key angiographic frame with sharp lumen contour and optimal exposure of the interrogated lesion(s); 2) the start and end points of the interrogated vessel for subsequent analysis. The above information was recorded by screenshots of each angiographic projection, with specific frame numbers, and markers for the start and end points.

The key frame in the first angiographic projection was used for μQFR1 analysis, while the key frame in the second angiographic projection was used for μQFR2 analysis. Both frames were used for 3D-μQFR analysis. The start and end points of each interrogated vessel were the same for μQFR1, μQFR2, and 3D-μQFR analyses.

# SUPPLEMENTAL FIGURES

## Supplemental Figure S1. Angiographic projection angle distribution.

**
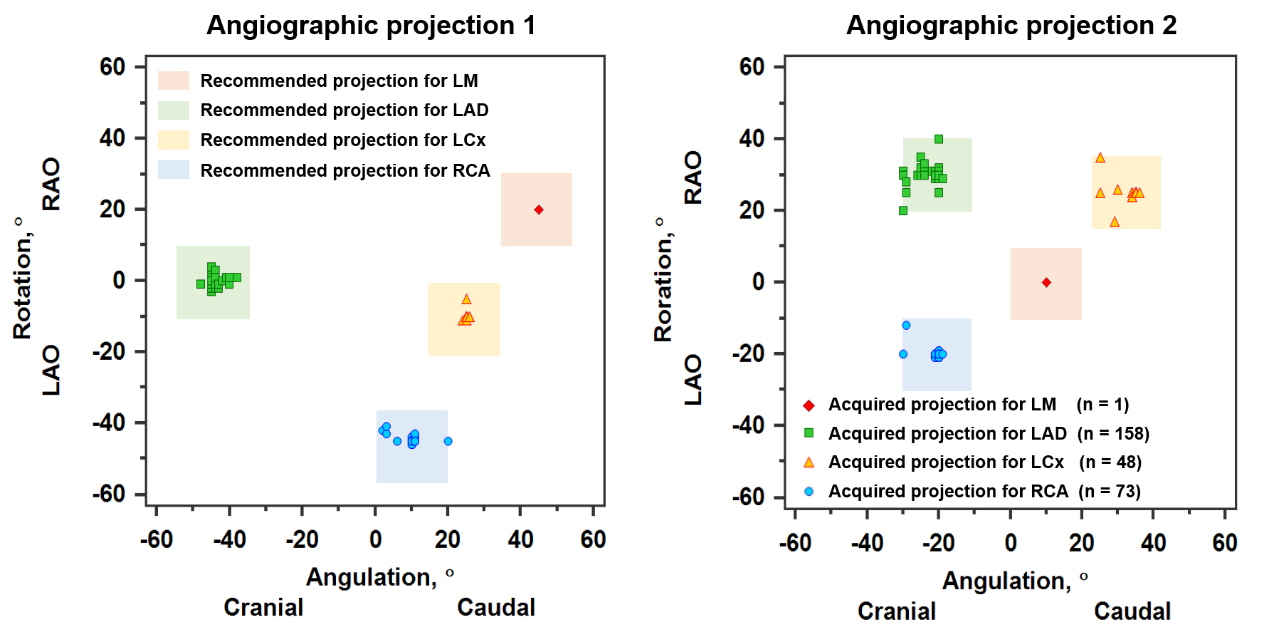
**

Vessels with both angiographic views acquired according to the protocol-specified recommended angiographic views used in FAVOR II China study were included into this study. We assumed that an angiographic projection was acquired according to the recommended angiographic view if the actual acquisition angle was within 10° deviation from the recommended angiographic projection. Red, green, yellow, and blue rectangulars demonstrate the range of recommended angiographic projection angles for interrogated lesions in LM, LAD, LCx, and RCA, respectively. The distribution of acquired angiographic projection angles is shown by dots of corresponding colors.

LAD = left anterior descending artery; LAO = left anterior oblique; LCx = left circumflex artery; LM = left main; RAO = right anterior oblique; RCA = right coronary artery.

## Supplemental Figure S2. Distribution histograms of FFR, μQFR1, μQFR2, and 3D-μQFR.


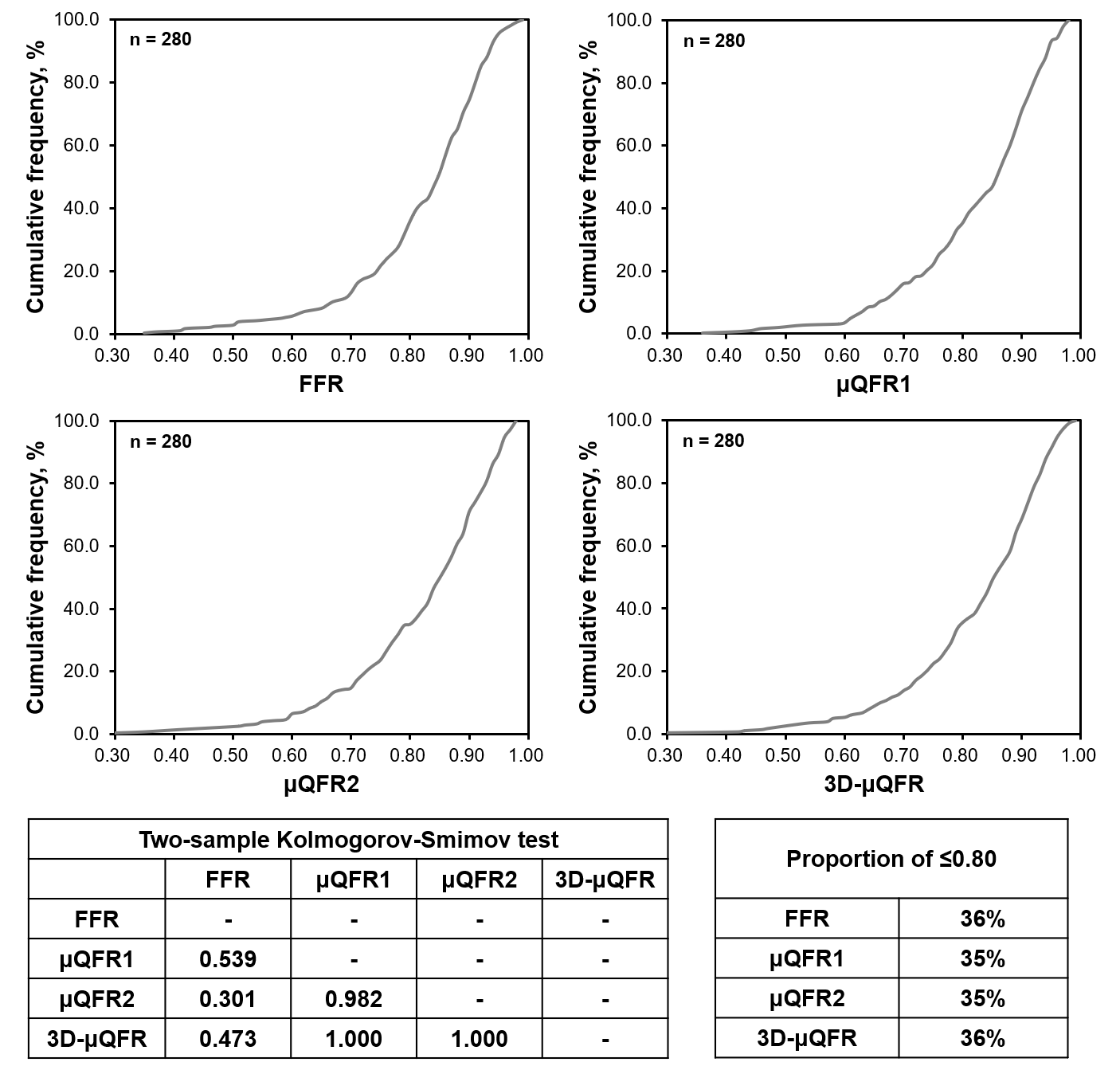


Two-sample Kolmogorov-Smimov test showed that distribution was similar for all paired comparison among FFR, μQFR1, μQFR2, and 3D-μQFR.

FFR = fractional flow reserve; μQFR = Murray law-based quantitative flow ratio; 3D = 3-dimensional.

# SUPPLEMENTAL TABLES

## Supplemental Table S1. Recommended optimal angiographic projections in FAVOR II China ^[1]^

| Interrogated Vessel | 1^st^ view | 2^nd^ view |
| --- | --- | --- |
| LM and LM bifurcation | 20º RAO, 45º Caudal | 0º LAO, 10º Caudal |
| LAD and diagonal artery | 0º LAO, 45º Cranial | 30º RAO, 20º Cranial |
| LCx and OM | 10º LAO, 25ºCaudal | 25º RAO, 35º Caudal |
| RCA/PLA/PDA | 45º LAO, 10º Caudal | 20º LAO, 20º Cranial |

LAD = left anterior descending artery; LAO = left anterior oblique; LCx = left circumflex artery; LM = left main; OM = obtuse marginal; PDA = posterior descending artery; PLA = postero-lateral branch; RAO = right anterior oblique; RCA = right coronary artery.

## Supplemental Table S2. Diagnostic concordance for predicting FFR ≤0.80

|  | | FFR | |  |
| --- | --- | --- | --- | --- |
|  |  | ≤0.80 | >0.80 |  |
| μQFR1 | ≤0.80 | **89** | **10** | 99 |
|  | >0.80 | **12** | **169** | 181 |
|  | | 101 | 179 | 280 |

|  | | FFR | |  |
| --- | --- | --- | --- | --- |
|  |  | ≤0.80 | >0.80 |  |
| μQFR2 | ≤0.80 | **89** | **9** | 98 |
|  | >0.80 | **12** | **170** | 182 |
|  | | 101 | 179 | 280 |

|  | | | FFR | | | |  | |
| --- | --- | --- | --- | --- | --- | --- | --- | --- |
|  |  |  | ≤0.80 | | >0.80 | |  |  |
| 3D-μQFR | ≤0.80 | | **91** | | **9** | | 100 | |
|  | >0.80 | | **10** | | **170** | | 180 | |
|  | | | 101 | | 179 | | 280 | |
|  | | | | FFR | | | |  |
|  |  |  |  | ≤0.80 | | >0.80 | |  |
| 2D-DS%1 | | ≥50% | | **50** | | **20** | | 70 |
|  |  | <50% | | **51** | | **159** | | 210 |
|  | | | | 101 | | 179 | | 280 |

|  | | FFR | |  |
| --- | --- | --- | --- | --- |
|  |  | ≤0.80 | >0.80 |  |
| 2D-DS%2 | ≥50% | **56** | **23** | 79 |
|  | <50% | **45** | **156** | 201 |
|  | | 101 | 179 | 280 |

|  | | FFR | |  |
| --- | --- | --- | --- | --- |
|  |  | ≤0.80 | >0.80 |  |
| 3D-DS% | ≥50% | **43** | **16** | 59 |
|  | <50% | **58** | **163** | 221 |
|  | | 101 | 179 | 280 |

2ⅹ2 Table for μQFR1, μQFR2, 3D-μQFR, and corresponding percent diameter stenosis versus FFR as reference standard on a per-vessel level. DS% = percent diameter stenosis; FFR = fractional flow reserve; μQFR = Murray law-based quantitative flow ratio; QCA = quantitative coronary angiography; SD = standard deviation; 2D = 2-dimensional; 3D = 3-dimensional.

## Supplemental Table S3. Discordances between μQFR1 and FFR using 0.80 as the cutoff value

| **Vessel** | | **No. of vessels** | **μQFR1** | | **FFR** | |
| --- | --- | --- | --- | --- | --- | --- |
|  |  |  | **Mean ± SD** | **Median (quartiles)** | **Mean ± SD** | **Median (quartiles)** |
| Overall | Overall | 22 | 0.80 ± 0.07 | 0.81 (0.78, 0.86) | 0.81 ± 0.05 | 0.80 (0.79, 0.84) |
|  | FN | 12 | 0.85 ± 0.04 | 0.84 (0.81, 0.87) | 0.78 ± 0.03 | 0.79 (0.77, 0.75) |
|  | FP | 10 | 0.75 ± 0.06 | 0.77 (0.72, 0.78) | 0.85 ± 0.03 | 0.85 (0.82, 0.88) |
| LAD | Overall | 16 | 0.80 ± 0.08 | 0.81 (0.77, 0.86) | 0.81 ± 0.04 | 0.80 (0.79, 0.83) |
|  | FN | 9 | 0.85 ± 0.04 | 0.86 (0.82, 0.87) | 0.78 ± 0.03 | 0.79 (0.78, 0.80) |
|  | FP | 7 | 0.73 ± 0.06 | 0.76 (0.70, 0.78) | 0.85 ± 0.03 | 0.84 (0.82, 0.88) |
| LCx | Overall | 1 | 0.72 | 0.72 | 0.89 | 0.89 |
|  | FN | 0 | \ | \ | \ | \ |
|  | FP | 1 | 0.72 | 0.72 | 0.89 | 0.89 |
| RCA | Overall | 5 | 0.82 ± 0.04 | 0.81 (0.80, 0.81) | 0.79 ± 0.05 | 0.80 (0.79, 0.82) |
|  | FN | 3 | 0.84 ± 0.05 | 0.81 (0.81, 0.89) | 0.77 ± 0.05 | 0.79 (0.71, 0.80) |
|  | FP | 2 | 0.80 ± 0.01 | 0.80 (0.79, 0.80) | 0.84 ± 0.02 | 0.84 (0.82, 0.85) |

FFR = fractional flow reserve; FN = false negative; FP = false positive; LAD = left anterior descending artery; LCx = left circumflex artery; RCA = right coronary artery; SD = standard deviation; μQFR = Murray law-based quantitative flow ratio.

## Supplemental Table S4. Discordances between μQFR2 and FFR using 0.80 as the cutoff value

| **Vessel** | | **No. of vessels** | **μQFR2** | | **FFR** | |
| --- | --- | --- | --- | --- | --- | --- |
|  |  |  | **Mean ± SD** | **Median (quartiles)** | **Mean ± SD** | **Median (quartiles)** |
| Overall | Overall | 21 | 0.81 ± 0.07 | 0.82 (0.78, 0.85) | 0.81 ± 0.04 | 0.80 (0.79, 0.84) |
|  | FN | 12 | 0.86 ± 0.04 | 0.85 (0.82, 0.90) | 0.78 ± 0.02 | 0.79 (0.78, 0.80) |
|  | FP | 9 | 0.75 ± 0.04 | 0.78 (0.71, 0.78) | 0.85 ± 0.03 | 0.85 (0.82, 0.88) |
| LAD | Overall | 15 | 0.82 ± 0.07 | 0.82 (0.78, 0.86) | 0.81 ± 0.05 | 0.80 (0.78, 0.84) |
|  | FN | 9 | 0.86 ± 0.04 | 0.85 (0.83, 0.87) | 0.78 ± 0.03 | 0.79 (0.78, 0.80) |
|  | FP | 6 | 0.75 ± 0.04 | 0.76 (0.71, 0.78) | 0.85 ± 0.03 | 0.86 (0.82, 0.88) |
| LCx | Overall | 1 | 0.68 | 0.68 | 0.89 | 0.89 |
|  | FN | 0 | \ | \ | \ | \ |
|  | FP | 1 | 0.68 | 0.68 | 0.89 | 0.89 |
| RCA | Overall | 5 | 0.82 ± 0.06 | 0.81 (0.79, 0.82) | 0.81 ± 0.02 | 0.80 (0.80, 0.82) |
|  | FN | 3 | 0.85 ± 0.06 | 0.82 (0.81, 0.92) | 0.80 ± 0.01 | 0.80 (0.79, 0.80) |
|  | FP | 2 | 0.79 ± 0.01 | 0.79 (0.78, 0.79) | 0.84 ± 0.02 | 0.84 (0.82, 0.85) |

FFR = fractional flow reserve; FN = false negative; FP = false positive; LAD = left anterior descending artery; LCx = left circumflex artery; RCA = right coronary artery; SD = standard deviation; μQFR = Murray law-based quantitative flow ratio.

## Supplemental Table S5. Discordances between 3D-μQFR and FFR using 0.80 as the cutoff value

| **Vessel** | | **No. of vessels** | **3D-μQFR** | | **FFR** | |
| --- | --- | --- | --- | --- | --- | --- |
|  |  |  | **Mean ± SD** | **Median (quartiles)** | **Mean ± SD** | **Median (quartiles)** |
| Overall | Overall | 19 | 0.80 ± 0.09 | 0.82 (0.75, 0.85) | 0.81 ± 0.05 | 0.80 (0.78, 0.84) |
|  | FN | 10 | 0.87 ± 0.04 | 0.85 (0.83, 0.91) | 0.77 ± 0.03 | 0.79 (0.76, 0.80) |
|  | FP | 9 | 0.73 ± 0.06 | 0.75 (0.71, 0.78) | 0.85 ± 0.03 | 0.84 (0.82, 0.88) |
| LAD | Overall | 15 | 0.80 ± 0.09 | 0.80 (0.75, 0.85) | 0.81 ± 0.05 | 0.81 (0.78, 0.84) |
|  | FN | 7 | 0.87 ± 0.05 | 0.85 (0.84, 0.91) | 0.77 ± 0.03 | 0.78 (0.76, 0.79) |
|  | FP | 6 | 0.74 ± 0.06 | 0.76 (0.72, 0.78) | 0.84 ± 0.03 | 0.83 (0.82, 0.88) |
| LCx | Overall | 1 | 0.68 | 0.68 | 0.89 | 0.89 |
|  | FN | 0 | \ | \ | \ | \ |
|  | FP | 1 | 0.68 | 0.68 | 0.89 | 0.89 |
| RCA | Overall | 3 | 0.86 ± 0.05 | 0.83 (0.83, 0.91) | 0.77 ± 0.05 | 0.80 (0.71, 0.80) |
|  | FN | 3 | 0.86 ± 0.05 | 0.83 (0.83, 0.91) | 0.77 ± 0.05 | 0.80 (0.71, 0.80) |
|  | FP | 0 | \ | \ | \ | \ |

FFR = fractional flow reserve; FN = false negative; FP = false positive; LAD = left anterior descending artery; LCx = left circumflex artery; RCA = right coronary artery; SD = standard deviation; μQFR = Murray law-based quantitative flow ratio; 3D = 3-dimensional.

## Supplemental Table S6. Diagnostic accuracy of μQFR1 for predicting FFR ≤0.80

| **Interrogated vessels** | **Accuracy** | |
| --- | --- | --- |
|  | Estimate, % (95% CI) | No. of vessels |
| LAD* | 90 (85, 95) | 159* |
| LCx | 98 (94, 100) | 48 |
| RCA | 93 (87, 99) | 73 |
|  | **Difference 95% (CI)** | **P value** |
| LAD vs. LCx | -8 (-14, 2) | 0.14 |
| LAD vs. RCA | -3 (-10, 6) | 0.62 |
| LCx vs. RCA | 5 (-5, 14) | 0.42 |

*Only one pair of angiographic projections were acquired according to the recommended angiographic views of left main, this vessel was included into the subgroup of LAD for comparative analyses.

CI = confidence interval; FFR = fractional flow reserve; LAD = left anterior descending artery; LCX = left circumflex artery; RCA = right coronary artery.

## Supplemental Table S7. Diagnostic accuracy of μQFR2 for predicting FFR ≤0.80

| **Interrogated vessels** | **Accuracy** | |
| --- | --- | --- |
|  | Estimate, % (95% CI) | No. of vessels |
| LAD* | 91 (86, 95) | 159* |
| LCx | 98 (94, 100) | 48 |
| RCA | 93 (87, 99) | 73 |
|  | **Difference 95% (CI)** | **P value** |
| LAD vs. LCx | -7 (-13, 7) | 0.38 |
| LAD vs. RCA | -2 (-9, 7) | 0.80 |
| LCx vs. RCA | 5 (-5, 14) | 0.42 |

*Only one pair of angiographic projections were acquired according to the recommended angiographic views of left main, this vessel was included into the subgroup of LAD for comparative analyses.

CI = confidence interval; FFR = fractional flow reserve; LAD = left anterior descending artery; LCX = left circumflex artery; RCA = right coronary artery.

## Supplemental Table S8. Diagnostic accuracy of 3D-μQFR for predicting FFR ≤0.80

| **Interrogated vessels** | **Accuracy** | |
| --- | --- | --- |
|  | Estimate, % (95% CI) | No. of vessels |
| LAD* | 91 (86, 95) | 159* |
| LCx | 98 (94, 100) | 48 |
| RCA | 96 (91, 100) | 73 |
|  | **Difference 95% (CI)** | **P value** |
| LAD vs. LCx | -7 (-13, 3) | 0.19 |
| LAD vs. RCA | -5 (-11, 3) | 0.28 |
| LCx vs. RCA | 2 (-8, 10) | 0.93 |

*Only one pair of angiographic projections were acquired according to the recommended angiographic views of left main, this vessel was included into the subgroup of LAD for comparative analyses.

CI = confidence interval; FFR = fractional flow reserve; LAD = left anterior descending artery; LCX = left circumflex artery; RCA = right coronary artery; 3D = 3-dimensional.

## Supplemental Table S9. Diagnostic accuracy for predicting FFR ≤0.80 in bifurcation lesions

| **Estimate, % (95% CI)** | **Bifurcation lesions (n=75)** | **Non-bifurcation lesions (n=205)** | **Difference** | **P value** |
| --- | --- | --- | --- | --- |
| μQFR1 | 93 (88, 99) | 92 (88, 96) | 0.02 (-0.06, 0.09) | 0.80 |
| μQFR2 | 93 (88, 99) | 92 (88, 96) | 0.01 (-0.06, 0.08) | 1.00 |
| 3D-μQFR | 95 (89, 100) | 93 (89, 96) | 0.02 (-0.05, 0.09) | 0.79 |

CI = confidence interval; FFR = fractional flow reserve; μQFR = Murray law-based quantitative flow ratio; 3D = 3-dimensional.

## Supplemental Table S10. Discordances among μQFR1, μQFR2, and 3D-μQFR

| **Case** | **Vessel** | **μQFR1** | **μQFR2** | **3D-μQFR** | **FFR** |
| --- | --- | --- | --- | --- | --- |
| Case 1 | RCA | 0.79 | 0.81 | 0.83 | 0.80 |
| Case 2 | LAD | 0.82 | 0.85 | 0.80 | 0.82 |
| Case 3 | LAD | 0.78 | 0.84 | 0.77 | 0.82 |
| Case 4 | RCA | 0.79 | 0.78 | 0.81 | 0.82 |
| Case 5 | LAD | 0.86 | 0.82 | 0.78 | 0.80 |
| Case 6 | RCA | 0.81 | 0.73 | 0.83 | 0.71 |
| Case 7 | RCA | 0.80 | 0.79 | 0.82 | 0.85 |
| Case 8 | LAD | 0.81 | 0.82 | 0.77 | 0.80 |
| Case 9 | RCA | 0.81 | 0.82 | 0.79 | 0.79 |

μQFR1, μQFR2 and 3D-μQFR values were concordant (all ≤0.80 or all >0.80) in 271 out of 280 vessels. The nine vessels (5 lesions in RCA and 4 lesions in LAD) with discordances among μQFR1, μQFR2 or 3D-μQFR values had a mean FFR of 0.80 ± 0.04 and median FFR of 0.80 [0.80 to 0.82].

FFR = fractional flow reserve; μQFR = Murray law-based quantitative flow ratio; 3D = 3-dimensional.

## Supplemental Table S11. Diagnostic performance of DS% in predicting FFR ≤0.80

|  | μQFR1-based DS% ≥50% | μQFR2-based DS% ≥50% |
| --- | --- | --- |
| Accuracy, % (95%CI) | 75 (70 – 80) | 76 (71 – 81) |
| Sensitivity, % (95%CI) | 50 (39 – 60) | 55 (45 – 65) |
| Specificity, % (95%CI) | 89 (88 – 93) | 87 (81 – 92) |
| PPV, % (95%CI) | 71 (59 – 82) | 71 (60 – 81) |
| NPV, % (95%CI) | 76 (69 – 81) | 78 (71 – 83) |
| +LR (95%CI) | 4.4 (2.8 – 7.0) | 4.3 (2.8 – 6.6) |
| -LR (95%CI) | 0.57 (0.5 – 0.7) | 0.51 (0.4 – 0.6) |
| AUC (95%CI) | 0.84 (0.78 – 0.88) | 0.82 (0.80 – 0.86) |
| Optimal cut-off by Youden index | ≥45% | ≥42% |

AUC = area under the ROC curve; CI = confidence interval; DS% = percent diameter stenosis; NPV = negative predictive value; PPV = positive predictive value; ROC = receiver operating characteristics; +LR = positive likelihood ratio; -LR = negative likelihood ratio; μQFR = Murray law-based quantitative flow ratio.

## Supplemental Table S12. Summary of key QFR validation studies

| **Study** | | | **Version** | Design | **Vessels** | **QFR Feasibility** | **FFR** | **Accuracy %** | **Sensitivity %** | **Specificity %** | **Compared to sensor position** | **Manual correction** |
| --- | --- | --- | --- | --- | --- | --- | --- | --- | --- | --- | --- | --- |
| Tu 2016 ^[2]^ | | | 1^st^ QFR | Prospective Offline | 84 | 73/89  (82%) | 0.84±0.08 | 86 | 74 | 91 | The QFR value at the position that matched the  location of the pressure transducer on the pressure wire was used for comparison with pressure wire-measured FFR value. | NR |
| Xu 2017 ^[1]^ | | | 1^st^ QFR | ProspectiveOffline | 328 | 329/332 (99%) | 0.82±0.12 | 93 | 95 | 92 | Before QFR analysis, the technicians were informed about the location where the operators intended to measure FFR so that QFR could be compared with FFR at the same vessel site. | NR |
| Yazaki 2017 ^[3]^ | | | 1^st^ QFR | RetrospectiveOffline | 151 | 131/151 (88%) | 0.84±0.07 | 89 | 89 | 89 | NR | NR |
| Ties 2018 ^[4]^ | | | 1^st^ QFR | RetrospectiveOffline | 101 | 101/333 (30%) | 0.87±0.08 | 90 | 67 | 96 | Setpoints for segment selection were placed at the location of the proximal tip of the FFR pressure sensor distally. | Aknowledged in Discussion that user interaction including manual correction of vessel contouring might affect QFR results |
| Mejía-Renteria 2018 ^[5]^ | | | 1^st^ QFR | RetrospectiveOffline | 300 | 300/381 (79%) | 0.80±0.11 | 88 | 89 | 87 | During QFR analysis, the distal point of the target vessel was indicated matching the original position of the pressure-wire sensor. | NR |
| Emori 2018 ^[6]^ | | MI (+) | 1^st^ QFR | RetrospectiveOffline | 75 | 150/163  (92%) | 0.79±0.11 | 87 | 92 | 82 | QFR was determined in all coronary arteries in which FFR was performed, at the location of the distal tip of the pressure-wire. | NR |
|  |  | MI (-) |  |  | 75 |  | 0.76±0.13 | 92 | 95 | 88 |  |  |
| Smit 2018 ^[7]^ | | | 1^st^ QFR | RetrospectiveOffline | 334 | 334/386 (87%) | 0.85±0.08 | 86 | 70 | 92 | NR | NR |
| Emori 2018 ^[8]^ | | | 1^st^ QFR | RetrospectiveOffline | 100 | 100/106 (94%) | 0.75±0.10 | 94 | 97 | 87 | NR | NR |
| Westra 2018 ^[9]^ | | | 1^st^ QFR | ProspectiveOffline | 240 | 240/295 (81%) | 0.82±0.11 | 83 | 77 | 86 | Pressure wire position was not documented angiographically. | NR |
| Spitaleri 2018 ^[10]^ | | | 1^st^ QFR | ProspectiveOffline | 49 | 49/49 (100%) | 0.84±0.11 | 94 | 88 | 97 | NR | NR |
| Westra 2018 ^[11]^ | | | 1^st^ QFR | ProspectiveOffline | 317 | 345/358 (96%) | 0.83±0.09 | 87 | 87 | 87 | The pressure transducer location was documented angiographically for all measurements. | NR |
| Koltowski 2018 ^[12]^ | | | 1^st^ QFR | RetrospectiveOffline | 306 | 306/857  (36%) | 0.80±0.10 | 85 | 84 | 87 | QFR value at the point corresponding to the pressure transducer on the FFR wire was recorded. | NR |
| Smit 2019 ^[13]^ | Diabetic | | 1^st^ QFR | RetrospectiveOffline | 82 | 320/372 (86%) | 0.85±0.08 | 88 | 71 | 95 | The QFR values on the coronary tree corresponded to the exact location of the pressure sensor at the angiogram. | NR |
|  | Non-diabetic | |  |  | 238 |  |  | 85 | 69 | 91 |  | NR |
| Stähli 2019 ^[14]^ | | | 1^st^ QFR | ProspectiveOffline | 516 | 516/575 (90%) | 0.88 [0.82–0.92] | 93 | 75 | 98 | The vessel segment used for vessel QFR analyses ends at the position of the pressure sensor located at the proximal end of the radiopaque wire tip. | NR |
| Hwang 2019 ^[15]^ | | | 1^st^ QFR | RetrospectiveOffline | 358 | 358/482 (74%) | 0.80±0.13 | 91 | 92 | 90 | NR | NR |
| Asano 2019 ^[16]^ | | | 1^st^ QFR | RetrospectiveOffline | 836 | 836/1177 (71%) | 0.78 [0.73–0.84] | 74 | 74 | 74 | QFR was analyzed to the anatomic site where iwFR and FFR were interrogated. | NR |
| Mejía-Rentería 2020 ^[17]^ | | | 1^st^ QFR | Retrospective Offline | 138 | 138/196 (70%) | 0.84 [0.77–0.89] | 81 | 84 | 80 | NR | NR |
| Tebaldi 2020 ^[18]^ | | | 1^st^ QFR | Prospective Online | 184 | 184/184 (100%) | 0.84±0.07 | 88 | 72 | 94 | NR | NR |
| Jin 2021 ^[19]^ | | | 1^st^ QFR | Retrospective Offline | 112 | 112/134 (84%) | 0.85±0.09 | 83 | 65 | 89 | The distal reference point for vessel reconstruction during QFR analysis was matched to the pressure wire sensor on coronary angiography. | The study compared QFR-derived QCA parameters with vs. without manual modifications |
| Tu 2021 ^[20]^ | | | 2^nd^ QFR | Retrospective Offline (angio prospectively acquired in FAVOR II China) | 330 | 330/330 (100%) | 0.82±0.12 | 93 | 88 | 96 | NR | NR |

NR = not reported; FFR = fractional flow reserve; iwFR = instantaneous wave-free ratio; QCA = quantitative coronary angiography; 1^st^ QFR = first generation of QFR solution; 2^nd^ QFR = second generation of QFR solution (i.e. Murray law-based QFR, μQFR).

# REFERENCES

1. Xu B, Tu S, Qiao S, et al. Diagnostic accuracy of angiography-based quantitative flow ratio measurements for online assessment of coronary stenosis. J Am Coll Cardiol 2017;70:3077-3087.

2. Tu S, Westra J, Yang J, et al. Diagnostic accuracy of fast computational approaches to derive fractional flow reserve from diagnostic coronary angiography: The international multicenter FAVOR Pilot study. J Am Coll Cardiol Intv 2016;9:2024-2035.

3. Yazaki K, Otsuka M, Kataoka S, et al. Applicability of 3-Dimensional Quantitative Coronary Angiography-Derived Computed Fractional Flow Reserve for Intermediate Coronary Stenosis. Circ J 2017;81:988-992.

4. Ties D, van Dijk R, Pundziute G, et al. Computational quantitative flow ratio to assess functional severity of coronary artery stenosis. Int J Cardiol 2018;271:36-41.

5. Mejía-Rentería H, Lee JM, Lauri F, et al. Influence of Microcirculatory Dysfunction on Angiography-Based Functional Assessment of Coronary Stenoses. JACC Cardiovasc Interv 2018;11:741-753.

6. Emori H, Kubo T, Kameyama T, et al. Diagnostic Accuracy of Quantitative Flow Ratio for Assessing Myocardial Ischemia in Prior Myocardial Infarction. Circ J 2018;82:807-814.

7. Smit JM, Koning G, van Rosendael AR, et al. Referral of patients for fractional flow reserve using quantitative flow ratio. Eur Heart J Cardiovasc Imaging 2019;20:1231-1238.

8. Emori H, Kubo T, Kameyama T, et al. Quantitative flow ratio and instantaneous wave-free ratio for the assessment of the functional severity of intermediate coronary artery stenosis. Coron Artery Dis 2018;29:611-617.

9. Westra J, Tu S, Winther S, et al. Evaluation of Coronary Artery Stenosis by Quantitative Flow Ratio During Invasive Coronary Angiography: The WIFI II Study (Wire-Free Functional Imaging II). Circ Cardiovasc Imaging 2018;11:e007107.

10. Spitaleri G, Tebaldi M, Biscaglia S, et al. Quantitative Flow Ratio Identifies Nonculprit Coronary Lesions Requiring Revascularization in Patients With ST-Segment-Elevation Myocardial Infarction and Multivessel Disease. Circ Cardiovasc Interv 2018;11:e006023.

11. Westra J, Andersen BK, Campo G, et al. Diagnostic performance of in‐procedure angiography‐derived quantitative flow reserve compared to pressure‐derived fractional flow reserve: The FAVOR II Europe‐Japan study. J Am Heart Assoc 2018;7:e009603.

12. Kołtowski Ł, Zaleska M, Maksym J, et al. Quantitative flow ratio derived from diagnostic coronary angiography in assessment of patients with intermediate coronary stenosis: a wire-free fractional flow reserve study. Clin Res Cardiol 2018;107:858-867.

13. Smit JM, El Mahdiui M, van Rosendael AR, et al. Comparison of diagnostic performance of quantitative flow ratio in patients with versus without diabetes mellitus. Am J Cardiol 2019;123:1722-1728.

14. Stähli BE, Erbay A, Steiner J, et al. Comparison of resting distal to aortic coronary pressure with angiography-based quantitative flow ratio. Int J Cardiol 2019;279:12-17.

15. Hwang D, Choi KH, Lee JM, et al. Diagnostic Agreement of Quantitative Flow Ratio With Fractional Flow Reserve and Instantaneous Wave-Free Ratio. J Am Heart Assoc 2019;8:e011605.

16. Asano T, Katagiri Y, Chang CC, et al. Angiography-Derived Fractional Flow Reserve in the SYNTAX II Trial: Feasibility, Diagnostic Performance of Quantitative Flow Ratio, and Clinical Prognostic Value of Functional SYNTAX Score Derived From Quantitative Flow Ratio in Patients With 3-Vessel Disease. JACC Cardiovasc Interv 2019;12:259-270.

17. Mejía-Rentería H, Nombela-Franco L, Paradis JM, et al. Angiography-based quantitative flow ratio versus fractional flow reserve in patients with coronary artery disease and severe aortic stenosis. EuroIntervention 2020;16:e285-e292.

18. Tebaldi M, Biscaglia S, Erriquez A, et al. Comparison of quantitative flow ratio, Pd/Pa and diastolic hyperemia-free ratio versus fractional flow reserve in non-culprit lesion of patients with non ST-segment elevation myocardial infarction. Catheter Cardiovasc Interv 2021;98:1057-1065.

19. Jin C, Ramasamy A, Safi H, et al. Diagnostic accuracy of quantitative flow ratio (QFR) and vessel fractional flow reserve (vFFR) estimated retrospectively by conventional radiation saving X-ray angiography. Int J Cardiovasc Imaging 2021;37:1491-1501.

20. Tu S, Ding D, Chang Y, Li C, Wijns W, Xu B. Diagnostic accuracy of quantitative flow ratio for assessment of coronary stenosis significance from a single angiographic view: A novel method based on bifurcation fractal law. Catheter Cardiovas Interv 2021;97:1040-1047.
